# WELL-BEING IN MIDDLE-AGED ADULTS: THE MEDIATING ROLE OF SOCIAL MEDIA USE

**DOI:** 10.1093/geroni/igad104.2740

**Published:** 2023-12-21

**Authors:** jingchuan wu, Xin Yao Lin, Tian Lin, David Almeida

**Affiliations:** Penn State University, State College, Pennsylvania, United States; Weill Cornell Medicine, New York City, New York, United States; University of Florida, Gainesville, Florida, United States; The Pennsylvania State University, State College, Pennsylvania, United States

## Abstract

As individuals age, social connection becomes increasingly important, and social media has the potential to facilitate social connections. This study aimed to investigate the relationship between age and well-being, focusing on the mediating role of social media use. While previous research has found social connection to be an important predictor of well-being, the specific implications of social media use for social connection on various aspects of well-being remain unclear. To explore this relationship, the study used publicly available data from the Midlife in the United States (MIDUS) study wave 3 and refresher 1, with a sample of 6871 participants (M = 56.80 yrs, SD = 14.58 yrs). Results showed that social media use played a significant mediating role in well-being, specifically in loneliness (b = 0.001, p=0.003), positive affect (b = -0.002, p< 0.001), and eudaimonic well-being (b = -0.020, p< 0.001). However, the indirect pathway from age to well-being through social media use was negative. That is, as individuals aged, they tended to use social media less frequently, which in turn was associated with worse well-being outcomes. These findings add to the expanding body of research on the complicated relationship between social media use and well-being, emphasizing the significance of focused interventions to encourage social media use for social connection in middle-aged adult populations. The study has implications for health professionals working with middle-aged adults, underlining the importance of considering social media use as a potential method of enhancing well-being.

